# Plasma incorporation, apparent retroconversion and β-oxidation of ^13^C-docosahexaenoic acid in the elderly

**DOI:** 10.1186/1743-7075-8-5

**Published:** 2011-01-27

**Authors:** Mélanie Plourde, Raphaël Chouinard-Watkins, Milène Vandal, Ying Zhang, Peter Lawrence, J Thomas Brenna, Stephen C Cunnane

**Affiliations:** 1 Research Center on Aging, Health and Social Sciences Center -- Sherbrooke University Geriatrics Institute, Department of Medicine, Université de Sherbrooke, Canada; 2 Faculté de Pharmacie, Centre de recherche du centre hospitalier de l'Université Laval (CHUQ), Québec, Canada; 3 Division of Nutritional Sciences, Cornell University, Ithaca, NY, USA

## Abstract

**Background:**

Higher fish or higher docosahexaenoic acid (DHA) intake normally correlates positively with higher plasma DHA level, but recent evidence suggests that the positive relationship between intake and plasma levels of DHA is less clear in the elderly.

**Methods:**

We compared the metabolism of ^13^C-DHA in six healthy elderly (mean - 77 y old) and six young adults (mean - 27 y old). All participants were given a single oral dose of 50 mg of uniformly labelled ^13^C-DHA. Tracer incorporation into fatty acids of plasma triglycerides, free fatty acids, cholesteryl esters and phospholipids, as well as apparent retroconversion and β-oxidation of ^13^C-DHA were evaluated 4 h, 24 h, 7d and 28d later.

**Results:**

Plasma incorporation and β-oxidation of ^13^C-DHA reached a maximum within 4 h in both groups, but ^13^C-DHA was transiently higher in all plasma lipids of the elderly 4 h to 28d later. At 4 h post-dose, ^13^C-DHA β-oxidation was 1.9 times higher in the elderly, but over 7d, cumulative β-oxidation of ^13^C-DHA was not different in the two groups (35% in the elderly and 38% in the young). Apparent retroconversion of ^13^C-DHA was well below 10% of ^13^C-DHA recovered in plasma at all time points, and was 2.1 times higher in the elderly 24 h and 7d after tracer intake.

**Conclusions:**

We conclude that ^13^C-DHA metabolism changes significantly during healthy aging. Since DHA is a potentially important molecule in neuro-protection, these changes may be relevant to the higher vulnerability of the elderly to cognitive decline.

## Background

The consumption of fish containing eicosapentaenoic (EPA) and docosahexaenoic (DHA) acids protects against cardiovascular disease risk [1] and is possibly associated with lower risk of cognitive decline [2,3]. Higher fish or higher EPA and DHA intake normally correlates positively with higher plasma EPA and DHA levels [4], but recent evidence suggests that the positive relationship between intake and plasma levels of EPA and DHA is less clear in the elderly [5-9]. For instance, EPA was 50 - 100% higher in plasma phospholipids (PL) and total lipids of the elderly than in young adults consuming the same EPA-enriched supplement [6-8]. Similarly, a DHA-enriched supplement increased DHA 42% more in plasma total lipids in healthy elderly compared to young adults [9]. Higher EPA and DHA concentrations is also reported in erythrocyte lipids of the elderly [10]. Several prospective epidemiological studies report that higher erythrocyte omega-3 fatty acids is associated with better cognitive function [11] or with lower risk of cognitive decline [12-16] in later life.

In the elderly, increased concentrations of omega-3 fatty acid in plasma significantly predict less cognitive decline over 3 years [17]. Unlike saturated and monounsaturated fatty acids, synthesis of DHA from its omega-3 precursor, alpha-linolenic acid, is extremely limited in humans [18]. Thus, it is recommended that DHA be obtained from dietary sources. DHA is a potential key molecule in neuro-protection because it is a major component of synaptic membranes and is involved in membrane repair and fluidity, cell signalling, initiation of anti-inflammatory processes and gene expression [19-22].

Tracing metabolism of carbon 13 (^13^C)-DHA may provide useful information about age-related changes in DHA metabolism in humans [23-25]. In young adults given an oral dose of 250 - 280 mg ^13^C-DHA, ^13^C enrichment peaked at 2 h post-dose in plasma triglycerides (TG) when the tracer was given in the TG form, but at 6 h post-dose when esterified to phosphatidylcholine [24,25]. Brossard et al. also reported 1.4% apparent retroconversion of ^13^C-DHA to ^13^C-docosapentaenoate (^13^C-DPA) and ^13^C-EPA 3 d after giving the tracer [24].

Neither the impact of aging on ^13^C-DHA metabolism nor β-oxidation of ^13^C-DHA has yet been investigated in humans but both may influence the somewhat higher blood EPA and DHA commonly seen in the healthy elderly [5-10]. Hence, our aim here was to evaluate the incorporation of ^13^C-DHA into plasma lipids, its apparent retroconversion to DPA and EPA, and its β-oxidation in a group of young adults ~27 y old versus a group of healthy elderly ~77 y old.

## Methods

All procedures reported here were approved by the Human Ethics Research Committee of the Health and Social Sciences Center -- Sherbrooke University Geriatrics Institute, which is the committee mandated to oversee human experimentation at our institution. All study participants gave informed written consent.

### ^13^C-DHA tracer study

Six young participants and six elderly were recruited (Table 1). All participants were non-smokers, were not pregnant or lactating, not using medication to control diabetes, liver disease, renal disease, hypertension, anemia or low serum albumin and had no other clinical evidence of malnutrition. An upper limit of 3.0% DHA in plasma total lipids was set to exclude those individuals who were probably already consuming fish oil supplements providing EPA and DHA or who were habitually eating relatively high amounts of fish. The participants' medical histories were taken by a registered nurse.

**Table 1:** Baseline characteristics (means ± SD) of the two groups

	Young (n = 6)	Elderly (n = 6)	p *
Age (years)	26.8 ± 2.6	76.5 ± 2.7	0.004
Male/Female	3/3	2/4	0.337
Weight (kg)	84.0 ± 20.1	64.9 ± 9.7	0.078
Body Mass Index (kg/m^2^)	26.9 ± 5.9	24.9 ± 3.5	0.670
Plasma Triglycerides (mmol/L)	0.71 ± 0.18	0.94 ± 0.28	0.078
Plasma Total Cholesterol (mmol/L)	4.32 ± 0.58	5.26 ± 0.33	0.016
			
Plasma Fatty Acids^a^(%)			
Sum Saturates	32.7 ± 2.1	29.3 ± 2.7	0.055
Sum Monounsaturates	24.6 ±3.2	26.6 ± 2.7	0.200
Linoleate	32.5 ± 5.0	30.9 ± 1.9	0.630
Arachidonate	6.3 ± 0.4	8.3 ± 1.3	0.013
α-Linolenate	< 0.1	0.5 ± 0.6	0.059
Eicosapentaenoate	0.6 ± 0.4	1.2 ± 0.3	0.012
Docosapentaenoate	0.1 ± 0.2	0.3 ± 0.3	0.211
Docosahexaenoate	1.7 ± 0.4	1.4 ± 0.8	0.872

^a^Plasma total lipids

*Mann-Whitney test

The ^13^C tracer used in this study was non-radioactive, safe for human research, uniformly labelled ( > 98%), and of high chemical purity [26]. At a breakfast scheduled in the Metabolic Unit of the Research Center on Aging, all participants received a muffin and a yogurt into which we added a single 50 mg dose of ^13^C-DHA as the methyl ester. The macronutrient composition of the 360 kCal muffin was: 13 g of fat containing 2 g saturated fat, 4.8 g of protein, 58.5 g of carbohydrates, 2.8 g of fibre and 28.9 g of sugar. Complete fatty acid profile of the muffin was not performed at the time of the experiment. The composition of the yogurt was 0 g of fat, 4 g of protein, 5.5 g of carbohydrates and 4.5 g of sugar for a total of 37.5 kCal. The composition of 200 mL orange juice provided 100 kCal, 1 g of protein, 23 g of carbohydrates and 20 g of sugar. The breakfast was consumed by all participants within 15 minutes after giving the tracer.

To follow appearance of ^13^CO2 coming from ^13^C-DHA β-oxidation, breath samples were collected at baseline and 4 h, 24 h and 7d after the tracer was consumed. To collect the breath samples, the participants breathed in a purpose-built device consisting of a perforated plastic bag attached to a mouthpiece (Easysampler, Quintron Instrument Company, Milwaukee, WI) where vacuum tubes were inserted to collect a sample of the expired breath [27,28]. Breath sample collection takes less than 30 sec. Our preliminary data showed that to effectively follow ^13^C-DHA metabolism in the blood, samples should be collected over a 4 wk period after tracer intake, by which time remaining ^13^C-DHA in plasma has essentially returned to baseline [29]. Enrichment of ^13^C-DHA in plasma lipid classes was performed by gas chromatography-combustion-isotope ratio mass spectrometry as previously described [30]. The ^13^C/^12^C values of the samples and the reference were used to calculate the δ per mil values which were designated thereafter as atom percent excess (APE). Calculation of ^13^C in DHA, DPA, EPA and α-linolenic acid (ALA) from APE values was done according to Brossard et al. [31]. Enrichment of ^13^C in breath CO2 following the ingestion of the ^13^C tracer was analyzed by isotope ratio mass spectrometry (Europa 20 - 20, Sercon Ltd, Crewe, Cheshire, UK) as previously described [28]. 5% CO2/N2 was the reference gas and He was the carrier gas (Praxair Canada Inc. Mississauga, ON, Canada). Percent dose recovered from β-oxidation of ^13^C-DHA in the breath was calculated as described by Freemantle *et al. *[27]. Cumulative oxidation of ^13^C-DHA was determined by calculating area under the curve between each time points using GraphPad Prism 5 software (San Diego, CA, USA) followed by summing the areas at each time.

### Analytical methodology

Total lipids from 0.5 ml plasma were extracted into chloroform-methanol and a mixture of esterified (TG, CE or PL) and non-esterified (FFA) heptadecanoate internal standard was added for quantification. The lipid classes were separated by thin layer chromatography using petroleum ether: ethyl ether: methanol: acetic acid (85:15:1:2.5) as migration solvent. At the end of the chromatographic runs, the plates were sprayed with a solution of 2',7'-dichlorofluorescein, and viewed under UV light. Free fatty acids (FFA) and TG were directly methylated with boron trifluoride in methanol (14%). Cholesteryl esters (CE) were saponified to remove cholesterol from the methyl esters mixture since cholesterol can damage our GC column if injected with our methyl esters. PL were saponified to assure full fatty acid recovery. Fatty acid recovery from TG is less of a problem than for PL so TG were directly transmethylated.

### Data expression and statistics

Six participants in each group was required to have sufficient power analysis with a β = 0.8 which is defined as 80% chance to reject the null hypothesis which state that there is no relationship between the variable of interest. Data are shown as means ± SEM, except as indicated. Sample sizes of less than 12 values simply don't provide enough data to discriminate between Gaussian and non-Gaussian distributions. In this situation non-parametric statistics are appropriate [32]. Fatty acid values and % β-oxidation were compared between young and elderly at each point using the non-parametric Mann-Whitney test (SPSS; Chicago, Illinois, USA). Apparent retroconversion of ^13^C-DHA was calculated by summing ^13^C-labelled DPA, EPA and ALA measured in plasma total lipids. The sum of ^13^C-labelled DPA, EPA and ALA was called ^13^C-omega-3 PUFA and was expressed as pmol/ml plasma. Statistical significance was set at p ≤ 0.05.

## Results

Mean age of the young and the elderly were 26.8 y and 76.5 y, respectively, so that there was a gap of 50 y between the two groups (Table 1). Weight and body mass index was not different between the two groups. At baseline, the elderly had 22% higher plasma cholesterol compared to the young. At baseline, the profile of fatty acids in total lipids of fasting plasma was similar between the young and the elderly with the exception of arachidonic acid and EPA, which were 32% and 100% higher in the elderly, respectively. At baseline, % DHA was not different between the two groups (Table 1).

### 13C-DHA in plasma

**Figure 1:**
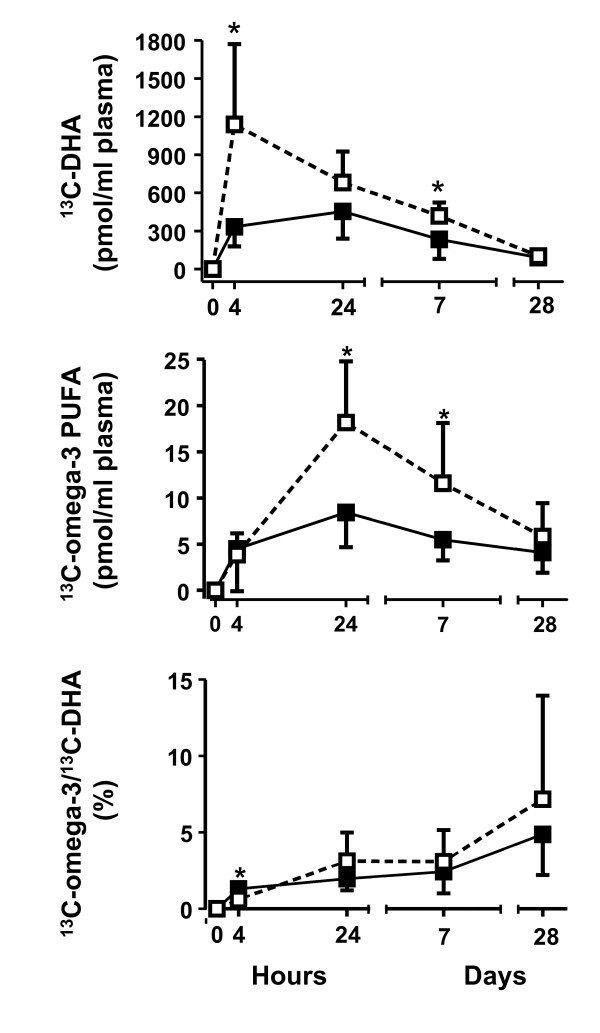
**^13^C-docosahexaenoic acid (DHA; upper panel), ^13^C-omega-3 PUFA (^13^C-alpha-linolenate + ^13^C-eicosapentaenoate + ^13^C-docosapentaenoate; middle panel) concentrations and ratio given as a percentage of ^13^C-omega-3 PUFA/^13^C-DHA (lower panel) in plasma total lipids of young (full symbols) and elderly (open symbols) over 28d after giving the ^13^C-DHA**. Data are shown for n = 5 for the young and n = 6 for the elderly (*P < 0.05).

Four h after giving the tracer, ^13^C-DHA peaked in plasma total lipids of the elderly (Figure 1, upper panel) at a level 3.5 times higher than in the young (p = 0.028). Plasma ^13^C-DHA concentration decreased 24 h after dosing, at which time it was no longer statistically different in the two groups. Seven days later, ^13^C-DHA concentration was still declining in both groups but was significantly higher in the elderly (p = 0.045). Twenty eight days post-intake, ^13^C-DHA returned close to baseline and was not different in the two groups (Figure 1A).

**Figure 2:**
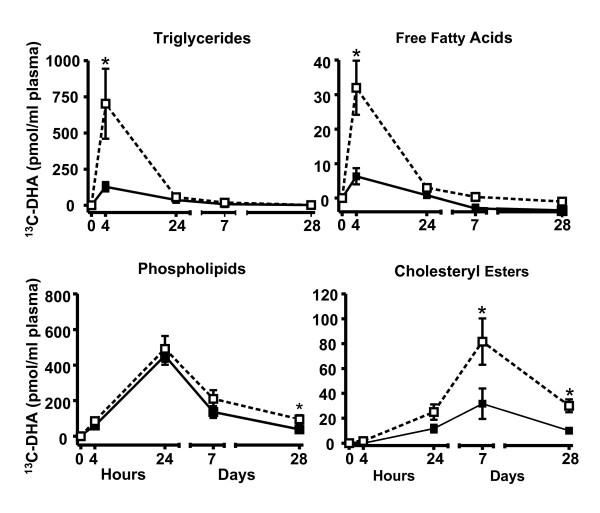
^13^C-docosahexaenoic acid (DHA) concentration in plasma triglycerides, free fatty acids, phospholipids, and cholesteryl esters in the young (full symbols) and the elderly (open symbols) followed over 28d (N = 6 for the young and the elderly except n = 4 for the elderly at 7d and 28d (* P < 0.05).

Four h post-dose, ^13^C-DHA was more than 4 fold higher in TG and FFA of the elderly but, in both groups, returned to near baseline after 24 h (Figure 2). ^13^C-DHA peaked in plasma PL 24 h post-dose but 28d later was still significantly higher in the elderly (p = 0.031; Figure 2). Seven days after dosing, ^13^C-DHA was 2.5 fold higher in CE of the elderly (p = 0.029) and remained so after 28d (p = 0.015).

### Apparent retroconversion (13C-omega-3 PUFA)

In both groups, the summed value for ^13^C-omega-3 PUFA other than DHA peaked 24 h after the dose of ^13^C-DHA and was 2.2 times higher in the elderly (p = 0.045; Figure 1, middle panel). ^13^C-omega-3 PUFA remained 2.1 times higher in the elderly 7d later. ^13^C-EPA contributed the most to the sum of ^13^C-omega-3 PUFA after 24 h (p = 0.045), whereas 7d later, ^13^C-EPA and ^13^C-ALA jointly contributed the most (data not shown). Percent apparent retroconversion expressed as a ratio of ^13^C-omega-3 PUFA relative to ^13^C-DHA concentration in plasma total lipids was not significantly different between the young and the elderly and reached 1.5 ± 1.1% and 2.0 ± 1.6%, respectively after 7d, and 2.5 ± 2.7% and 4.3 ± 6.2% after 28d, respectively (Figure 1, lower panel).

### β-Oxidation

**Figure 3:**
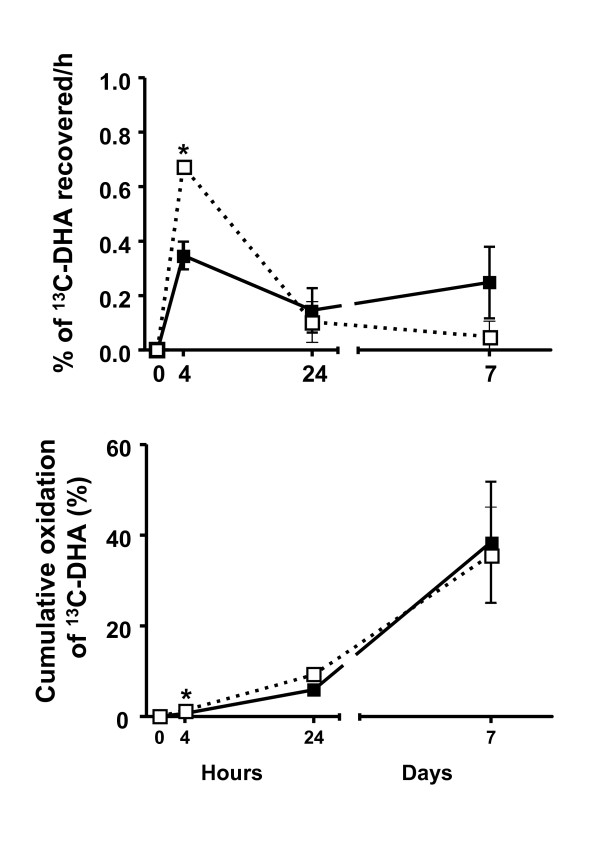
β-oxidation of ^13^C-docosahexaenoic acid (^13^C-DHA) shown as % dose recovered over time (upper panel) and as cumulative β-oxidation over time (lower panel) in young (full symbols) and the elderly (open symbols) over 7d (n = 6 for the young and the elderly except n = 4 for the elderly at 24 h; *P < 0.05).

Carbon-13 values in breath samples at 28d were very low and not available in 3 young participants and 5 elderly participants. Hence, % dose of ^13^C-CO2 and cumulative β-oxidation of ^13^C-DHA was calculated over the first 7d post-dose (Figure 3). As a % of the dose given, ^13^C-DHA β-oxidation was 1.9 times higher at 4 h post-dose in the elderly (p = 0.004), but was not significantly different between the young and the elderly at 24 h or at 7d (Figure 3; upper panel). Cumulative β-oxidation over 7d reached 35% and 38% in the elderly and the young participants, respectively (not different; Figure 3, lower panel).

## Discussion

The aim of this study was to evaluate whether plasma incorporation, apparent retroconversion, or β-oxidation of ^13^C-DHA differed in the healthy elderly compared to healthy young adults. We observed that within the post-prandial period (+ 4 h) the elderly had at least 4 fold higher ^13^C-DHA in plasma TG and FFA, and 1.9 fold higher β-oxidation to ^13^C-CO_2_. ^13^C-DHA was also 2.5 fold higher in plasma CE of the elderly after 7d, and its apparent retroconversion was 2.2 fold higher in the elderly 24 h after the oral dose. Hence, our results indicate that there are significant differences in DHA metabolism during healthy aging which compliment, extend and possibly help explain previous reports showing somewhat higher plasma DHA in the elderly [5-9].

The distribution of ^13^C-DHA we observed in plasma lipid classes of our young study participants agrees with previous published papers [24,25,31]. We speculate that the higher early rise in ^13^C-DHA in plasma TG and FFA in the elderly (Figure 2) may potentially be explained by the elderly having both higher postprandial production of very low density lipoproteins which are rich in TG [33], as well as a higher post-prandial FFA response [7]. However, one limitation is that postprandial TG-rich lipoproteins were not measured in this study so this explanation remains to be confirmed. The 4-fold higher ^13^C-DHA in plasma CE in the elderly after 7d persisted out to 28d and may be due to different lipoprotein metabolism in the elderly as suggested by their (i) higher plasma total cholesterol (Table 1), (ii) higher plasma residence time of low density lipoprotein [34], and/or (iii) lower turnover of low density lipoprotein [33].

The appearance of ^13^C in other omega-3 PUFA besides DHA (mostly in ^13^C-EPA) was about 2 times higher in the elderly 24 h to 7d after tracer intake. This measure of apparent retroconversion of ^13^C-DHA to ^13^C-EPA could occur by one cycle of β-oxidation and chain shortening [35]. Subsequent chain lengthening of ^13^C-EPA could explain the appearance of ^13^C-DPA. Alternatively, recycling of carbon from PUFA through ^13^C-acetate into newly synthesized fatty acids is a well known phenomenon [35], and could potentially become part of ALA because humans can make small amounts of ALA from 16:3 n-3, or omega-3 PUFA longer than ALA via conventional elongation [36].

The mean total ^13^C-EPA and ^13^C-DPA appearing in plasma is well below 10% even at 28d post-dose, so this amount of apparent retroconversion would seem unlikely to fully account for net > 5% rise in plasma phospholipid EPA after DHA supplementation reported elsewhere [37], unless the process were substantially up-regulated using multigram DHA supplements. Raised plasma EPA after a DHA supplement may also be due to reduced EPA turnover by sparing mechanisms with enhanced dietary DHA.

We show for the first time that ^13^C-CO_2 _from β-oxidation of ^13^C-DHA peaked at 4 h post-intake and was 1.9 fold higher in the elderly (Figure 3). These data confirm a preliminary report [29] showing that humans β-oxidize ^13^C-DHA much more slowly than ^13^C-ALA [28]. The major regulatory mechanism for the control of β-oxidation is the availability of substrate, mostly as fatty acids in postprandial circulating TG and FFA [38]. The higher β-oxidation of ^13^C-DHA at 4 h post-dose in our elderly participants is in line with their higher ^13^C-DHA in plasma TG and FFA 4 h post-dose (Figure 2). After 7d, ^13^C-CO2 was still above baseline in both groups although plasma ^13^C-DHA concentration had returned close to baseline in TG and FFA. Cumulative β-oxidation of ^13^C-DHA was not different in the young and the elderly and reached 35 - 38% after 7d, which is about half that seen for the same dose of ^13^C-ALA over the same time period [28]. Under the conditions of this study, these results give a rough estimate of about 3 wk to turnover DHA to expired CO2, or a DHA biological half-life of about 10 d. However, overall β-oxidation was not higher in the elderly even though the elderly did have ^13^C-EPA derived from ^13^C-DHA. There is no implicit reason why the age-related difference in recovery of ^13^C as ^13^C-EPA should be directly related to net loss of ^13^C-DHA to ^13^CO_2_; both processes involve a complex interchange of carbon. Furthermore, we do not yet know how the EPA and other omega-3 fatty acids became labelled with ^13^C; if it was via ‘direct’ retroconversion, i.e. by chain shortening, there is no implicit reason why this form of retroconversion should be coupled to the β-oxidation of DHA; if it was via oxidation to acetyl-CoA, perhaps one would expect a closer correlation between β-oxidation of DHA and EPA labelling. Analytically, we cannot yet distinguish between these two possibilities. Incidentally, the elderly did have higher β-oxidation, but only at the earliest time point (+ 4 h).

The possible relevance of aging-related changes in DHA metabolism to risk of chronic diseases, particularly cognitive decline, remains to be established. Fish intake seems to decrease the risk of cognitive decline [2] and EPA and DHA in blood are biomarkers of fish intake [4] but, paradoxically, an overview of the literature shows that lower blood DHA is not seen in Alzheimer's disease or other forms of dementia [2,39]. Indeed, the aging-associated changes in DHA metabolism we report here suggest that higher plasma DHA in the elderly could actually mask lower DHA availability to the brain. Another factor that was not considered in this small study is the presence of apolipoprotein E ε4 gene polymorphisms which appear to alter plasma EPA and DHA metabolism [40].

Because of the limited availability of the tracer and its very high cost, we had only six participants per group. However, a tracer is much more sensitive and specific than a fish oil supplement such that low number of participants per group can still generate useful and statistically valid results [23]. Indeed, previous studies using ^13^C-DHA used only 3 - 4 participants [24,25,29,31].

## Conclusions

We conclude from this month long study that in the healthy elderly, 50 mg of ^13^C-DHA is retained longer in the blood and undergoes more β-oxidation and more apparent retroconversion than in young adults. Given the potential importance of DHA for cardiovascular and brain health and the increasing elderly population, this alteration in DHA metabolism in the elderly may have some bearing on their vulnerability to cognitive decline. Further studies with ^13^C-DHA should examine -- (i) mechanisms at the root of these aging-associated differences, (ii) whether there is a link to the cognitive health of the elderly, and (iii) whether the dietary need for DHA may be different in the elderly.

### Abbreviations

ALA: α-linolenic acid; CE: cholesteryl esters; DHA: docosahexaenoic acid; DPA: docosapentaenoic acid; EPA: eicosapentaenoic acid; FFA: free fatty acids; TG: triglycerides

### Competing interests

The authors declare that they have no competing interests.

### Authors' contributions

MP, MV and SCC designed the study; MP, RCW and MV conducted the study; YZ, PL and JTB conducted the 13C-analyses; MP, RCW, MV, JTB and SCC analyzed the data, and MP and RCW performed the statistical analysis. MP prepared the first draft and all authors contributed to writing and reviewing the paper, MP had primary responsibility for final content. All authors read and approved the final manuscript.
